# Prognostic role of “prion-like propagation” in SOD1-linked familial ALS: an alternative view

**DOI:** 10.3389/fncel.2014.00359

**Published:** 2014-10-31

**Authors:** Keizo Sugaya, Imaharu Nakano

**Affiliations:** Department of Neurology, Tokyo Metropolitan Neurological HospitalTokyo, Japan

**Keywords:** aggregate, amyotrophic lateral sclerosis, mutation, nucleation, prion, SOD1, kinetic model

## Abstract

“Prion-like propagation” has recently been proposed for disease spread in Cu/Zn superoxide dismutase 1 (SOD1)-linked familial amyotrophic lateral sclerosis (ALS). Pathological SOD1 conformers are presumed to propagate via cell-to-cell transmission. In this model, the risk-based kinetics of neuronal cell loss over time appears to be represented by a sigmoidal function that reflects the kinetics of intercellular transmission. Here, we describe an alternative view of prion-like propagation in SOD1-linked ALS – its relation to disease prognosis under the protective-aggregation hypothesis. Nucleation-dependent polymerization has been widely accepted as the molecular mechanism of prion propagation. If toxic species of misfolded SOD1, as soluble oligomers, are formed as on-pathway intermediates of nucleation-dependent polymerization, further fibril extension via sequential addition of monomeric mutant SOD1 would be protective against neurodegeneration. This is because the concentration of unfolded mutant SOD1 monomers, which serve as precursor of nucleation and toxic species of mutant SOD1, would decline in proportion to the extent of aggregation. The nucleation process requires that native conformers exist in an unfolded state that may result from escaping the cellular protein quality control machinery. However, prion-like propagation-SOD1 aggregated form self-propagates by imposing its altered conformation on normal SOD1-appears to antagonize the protective role of aggregate growth. The cross-seeding reaction with normal SOD1 would lead to a failure to reduce the concentration of unfolded mutant SOD1 monomers, resulting in continuous nucleation and subsequent generation of toxic species, and influence disease prognosis. In this alternative view, the kinetics of neuronal loss appears to be represented by an exponential function, with decreasing risk reflecting the protective role of aggregate and the potential for cross-seeding reactions between mutant SOD1 and normal SOD1.

## INTRODUCTION

Amyotrophic lateral sclerosis (ALS) is a progressive, fatal neurodegenerative disease affecting motor neurons for which there is no effective treatment. The average disease duration is about 3 years, but it can vary significantly. Death usually results from compromised function of respiratory muscles. Since the landmark discovery in 1993 that mutations in the Cu/Zn superoxide dismutase 1 (SOD1) gene cause the familial form of ALS (FALS; Rosen et al., 1993), the underlying molecular features, and associated clinical characteristics of this disease have been extensively annotated. More than 150 different mutations of SOD1 have been found in patients with familial ALS. Studies on the clinical course of FALS suggest that the duration of illness is relatively consistent for each SOD1 mutation, but variable among the different mutations; for example, patients with the A4V mutation survive an average of 1 year after diagnosis, whereas patients with the H46R mutation survive an average of 18 years (Czaplinski et al., 2006). Aggregation of misfolded SOD1 proteins is a common pathological finding among subjects with different SOD1 mutations and is therefore believed to be central to disease pathogenesis (Johnston et al., 2000; Kato et al., 2001; Wang et al., 2003). The severity of mutant-induced destabilizing effects on the SOD1 molecule appears to correlate weakly with disease progression (Lindberg et al., 2005; Sato et al., 2005). However, the molecular mechanisms underlying variations in age of onset and disease duration of these conditions remain largely unknown.

Using ALS models derived from human embryonic stem cells or transgenic mice, recent studies have demonstrated that both cell-autonomous and non-cell-autonomous processes contribute to neurodegeneration in SOD1-linked FALS (Nagai et al., 2007; Di Giorgio et al., 2007; Yamanaka et al., 2008; Wada et al., 2012). Mutant SOD1 overexpression in differentiated motor neurons is sufficient to induce selective cell death and formation of aggregates as inclusions that mimic the* in vivo* human ALS disease. Furthermore, neuronal losses are enhanced when motor neurons are co-cultured with astrocytes expressing mutant SOD1 (Nagai et al., 2007; Di Giorgio et al., 2007). Clinically, the relationship between mean age at onset and mean survival time among patients with different SOD1 mutations are poorly correlated (Sato et al., 2005; Wang et al., 2008). These findings suggest differences in the mechanisms responsible for the initiation and progression of the neurodegenerative process in SOD1-linked FALS.

Prion disorders, such as Creutzfeldt-Jakob disease, are infectious diseases caused by the amyloid form of the prion protein, PrP^Sc^, which endlessly self-propagates by imposing its altered conformation on the cellular prion protein, PrP^C^ (Prusiner, 1998). We use the term “prion-like propagation” to describe this molecular event—self-templating replication to cross-seed aggregation of normal cellular counterparts. Recent studies have suggested prion-like propagation as a mechanistic model of lesion spread in SOD1-linked FALS (Grad et al., 2011; Munch et al., 2011; Polymenidou and Cleveland, 2011; Grad and Cashman, 2014). Although there has been no evidence of transmission of SOD1 aggregates between individuals, using cultured neuronal cells, mutant SOD1 aggregates showed prion-like behavior, a process involving a cross-seed aggregation of normal SOD1 and cell-to-cell transmission of misfolded SOD1 aggregates (Grad et al., 2011, 2014; Munch et al., 2011). Neurodegeneration in ALS typically begins focally and then spreads spatiotemporally until motor neurons of the respiratory system are lost (Ravits et al., 2007a,b). An attractive model for this progression would be the spread of toxic aggregates from a focal site through cell-to-cell transmission.

Aggregation of misfolded proteins is a pathological hallmark of many neurodegenerative diseases and is generally considered to be controlled by nucleation-dependent polymerization—a two stage processes consisting of the energetically unfavorable formation of a nucleus (i.e., nucleation), followed by efficient elongation of that nucleus via sequential addition of monomers. There is a major controversy concerning the role of aggregates growth in disease pathogenesis. One hypothesis is that these aggregates play a vital role in both disease initiation and progression, with the misfolded versions of endogenous proteins likely to acquire toxic properties, potentially through increased hydrophobicity and/or sequestration of essential cellular components within the aggregates and other pathways. An alternative hypothesis is that the large aggregates represent not the toxic species but rather the final product of a defensive response aimed at protecting cells from more toxic oligomeric species.

Under the assumption that toxic species of misfolded SOD1, as soluble oligomers, are formed as on-pathway intermediates of nucleation-dependent polymerization, we describe here an alternative view of prion-like propagation in SOD1-related ALS mechanism. These different notions reflect uncertainties surrounding the roles of misfolded protein aggregates (toxic versus protective). In this alternative view, the ability of the aggregated form of SOD1 to cross-seed aggregate normal SOD1 appears to antagonize the protective role of aggregate growth. Thus, prion-like propagation would be expected to exert a significant influence on disease prognosis. On the basis of available data for SOD1-linked FALS, together with a risk-based modeling approach, we show that prion-like propagation may account for up to 84% of the variability in survival times among subjects with different SOD1 mutations, highlighting the prognostic value of prion-like propagation and providing potential therapeutic targets for SOD1-linked FALS.

## PRION-LIKE PROPAGATION IN SOD1-LINKED FALS

**FIGURE 1:**
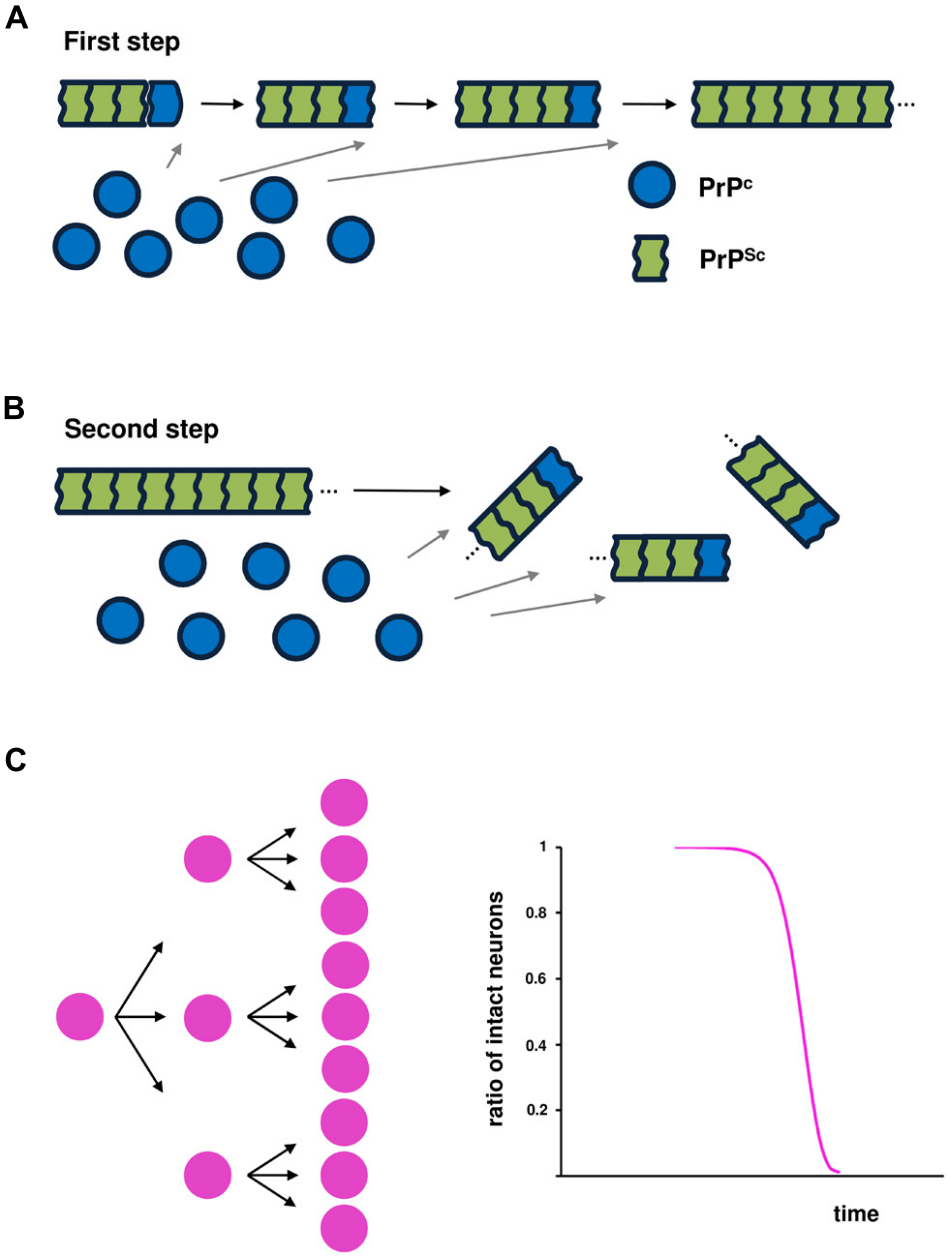
**Prion propagation based on nucleation-dependent polymerization.** Two events are essential for infectivity: **(A)** conversion of the α-helix-rich, host-encoded prion protein (PrP^C^) into a pathogenic conformer (PrP^Sc^), characterized by a higher β-sheet content and a polymeric state, through a nucleation-dependent polymerization mechanism; and **(B)** fragmentation of the PrP^Sc^ amyloid conformer to liberate new fibril ends that are the sites of the conversion of PrP^C^ into PrP^Sc^, leading to amplification of conformational replication. **(C)** Schematic illustration of the kinetics of infected cells over time.

Two essential steps are required for prion infectivity: self-templating replication and an efficient replication cycle (Cushman et al., 2010). For efficient infectivity, pathogenic prion proteins overwhelm the first key step of self-templating replication by converting the α-helix-rich host-encoded PrP^C^ into PrP^Sc^, characterized by a higher β-sheet content and a polymeric state (**Figure 1A**; Prusiner, 1998; Jackson et al., 1999). This process is thought to be achieved by a nucleation-dependent polymerization mechanism. Typically, this capture and conversion process requires that native conformers exist in a transiently unfolded state or possess an intrinsically unfolded domain. The second step is associated with frangibility of the fibril structure. Amplification of conformational replication is achieved by the fragmentation of amyloid forms to liberate new fiber ends where prion replication occurs. Increased frangibility leads to more fiber ends per unit mass and, consequently, more rapid conversion of available monomers (**Figure 1B**; Tanaka et al., 2006; Colby et al., 2009).

Cu/Zn superoxide dismutase 1 aggregated form could also seed misfolding of a much larger amount of wild-type (WT) SOD1* in vitro* (Chia et al., 2010). Moreover, in cultured cells, SOD1 aggregated forms, either exogenously applied (Munch et al., 2011; Grad et al., 2014) or formed within cells (Grad et al., 2011), induced the misfolding and subsequent aggregation of the respective native proteins. Importantly, induced aggregation of endogenous SOD1 was shown to persist after removal of the misfolded seeds, suggesting that the newly formed aggregates could act as templates for the subsequent misfolding of additional native SOD1 (Grad et al., 2011, 2014; Munch et al., 2011). Although fragmentation of the SOD1 aggregated forms to liberate new fiber ends has not yet been verified, these behaviors are consistent with a self-perpetuating, cyclic reaction, analogous to that underlying the replication of infectious prion aggregates. These findings raise the possibility that SOD1-linked ALS progresses through the spread of toxic SOD1 aggregates from a focal site in a manner similar to that of prion spread (cell-to-cell transmission of pathological misfolded prions). However, studies of SOD1 have found no evidence that directly links the transmission of SOD1 aggregates to neuronal toxicity.

Work over the past 4 years indicates that multiple proteins associated with neurodegenerative diseases, especially tau and α-synuclein can propagate aggregates between cells in a prion-like manner. Frost et al. (2009) demonstrated, for the first time, that aggregates of tau protein were taken up into cultured cells where they could induce fibrillization of intracellular tau (Guo and Lee, 2011). Further, tau aggregates newly formed in a cell were observed to transfer to co-cultured cells (Frost et al., 2009). This work was subsequently replicated for α-synuclein (Desplats et al., 2009; Luk et al., 2009; Lee et al., 2010; Hansen et al., 2011; Volpicelli-Daley et al., 2011; Freundt et al., 2012) and SOD1(Munch et al., 2011). It is now well established that protein aggregates are mobile, and can transmit aggregates from cell to cell *in vitro*.

### KINETICS OF NEURONAL CELL LOSS

In the model of prion-like propagation in SOD1-linked FALS (cell-to-cell transmission of pathological SOD1 conformers), it is predicted that the kinetics of neuronal cell death over time can be expressed by a sigmoidal function that reflects the kinetics of intercellular transmission over time (**Figure 1C**). If one amyloid conformer of a disease-specific protein liberates three particles that can penetrate a cell, then after fibril growth, the next fragmentation would yield nine particles. Thus, the risk of intercellular transmission would be accelerated in early and middle stages of the disease through fragmentation of the amyloid conformer, but the efficiency of transmission would decline in the advanced stage.

## AN ALTERNATIVE VIEW OF PRION-LIKE PROPAGATION IN SOD1-LINKED FALS

Under the assumption that toxic species of disease-specific proteins, for example as soluble oligomers, are formed as on-pathway intermediates of fibril growth through nucleation-dependent polymerization, further fibril extension would be protective against neurodegeneration. According to the free energy profile as a function of aggregate size, free energy peaks at the nucleus stage, and decreases in proportion to the extent of growth of aggregates (Oosawa and Kasai, 1962). Thus, toxic species of misfolded proteins are expected to be structurally stabilized. Importantly, an additional protective effect of aggregate growth is to reduce the concentration of the unfolded/misfolded monomeric proteins. These species of disease-specific proteins are considered to serve as precursors of nucleation—a critical stage in the assembly of a polymeric structure. The fibril nucleation rate is determined as an explicit function of the concentration of the protein solution (Ferrone, 1999). The rate constants for nucleation, estimated by non-linear least-squares algorithms, are ∼10,000,000 times smaller than those for fibril growth (Lee et al., 2007). A reduction in the corresponding protein concentration inhibits the nucleation process and subsequent generation of the toxic species of disease-specific proteins in the affected cells.

In contrast to prion-like propagation in disease spread, we present an alternative view of prion-like propagation in the context of the protective-aggregation hypothesis in which SOD1 toxic species are formed as an on-pathway intermediate in nucleation-dependent polymerization. Although the cross-seeding reaction of prion-like propagation seems to have little influence on the stabilizing effect of fibril growth on the toxic species of misfolded SOD1, it can be predicted to antagonize the protective role of aggregate growth.

**FIGURE 2:**
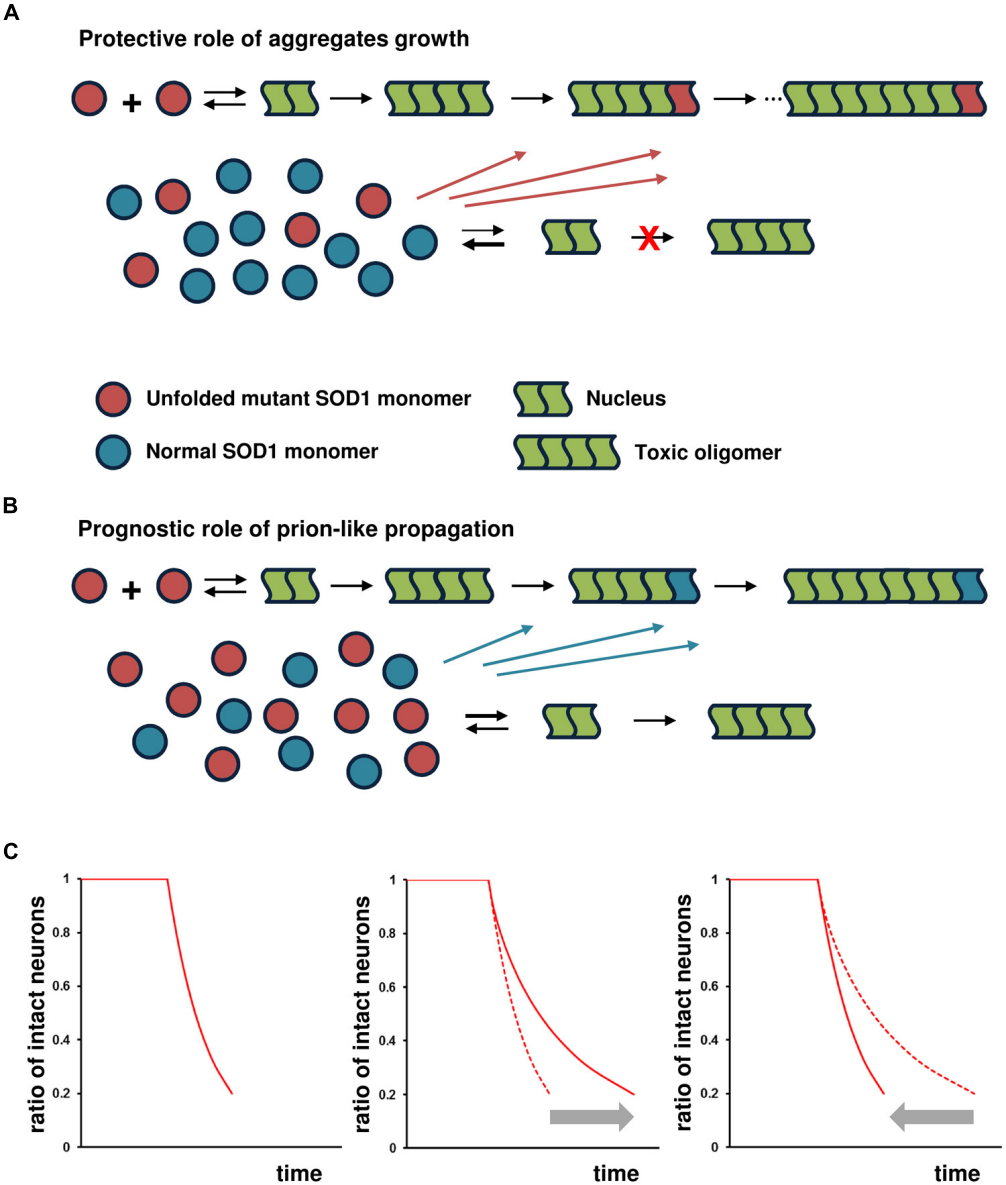
**Prognostic role of prion-like propagation in SOD1-linked FALS.** Under the assumption that toxic species of misfolded SOD1 are formed via on-pathway nucleation-dependent polymerization, prion-like propagation antagonizes the protective role of aggregate growth. Small oligomeric forms may be toxic, but for the sake of simplicity, the toxic species are shown as tetramers in this schema. The number of monomeric units involved in the formation of the nucleus for SOD1 aggregation is still unknown, but shown as a dimer in this schema. **(A)** SOD1 aggregate growth decreases the probability of the next generation of nucleation and formation of toxic species by reducing the concentration of unfolded mutant SOD1 monomers in the affected cells (protective role of aggregate growth). **(B)** The ability of the SOD1 aggregated form to cross-seed with normal SOD1 results in a failure to reduce the concentration of unfolded mutant SOD1 monomers, leading to continuous nucleation and the subsequent generation of toxic species (prognostic role of prion-like propagation). **(C)** Under the assumption of constant risk for neuronal cell damage, the kinetics of neuronal loss (proportion of intact neurons as a function of time) shows a first-order exponential function (left panel). The kinetics of cell death shows an exponential function with decreasing risk, with further elongation of aggregates reducing the risk of cell death in proportion to the extent of the increase in aggregate size (middle panel). The significant influence of prion-like propagation on disease prognosis through cross-seed aggregation of normal SOD1 would closely fit the exponential function indicated by the gray arrow (right panel).

Instead of cell-to-cell transmission of the pathological SOD1 conformer, the endogenous, stochastic occurrence of mutant SOD1 nucleation in neuronal cells plays a pivotal role in disease initiation and spread. The subsequent generation of toxic species of SOD1 then causes damage to the cells. In the affected cells, however, further aggregate growth decreases the probability of the next generation of nucleation by reducing the concentration of unfolded mutant SOD1 monomers into the fibril under the protective-aggregation hypothesis (**Figure 2A**). This species of SOD1 is considered to serve as a precursor of nucleation and subsequent toxic species (Furukawa et al., 2004; Hough et al., 2004; Schmidlin et al., 2009). However, if the SOD1 aggregated form preferentially self-propagates by cross-seeding aggregates of normal SOD1 monomers; the result would be a failure to reduce the concentration of unfolded mutant SOD1 monomers, leading to continuous nucleation and the generation of toxic species in the affected cells (**Figure 2B**). Thus, it is assumed that the ability of the reaction underlying prion-like propagation to cross-seed normal SOD1 exerts a significant influence on disease prognosis. The concentration of unfolded mutant SOD1 monomers varies according to the degree of the cross-seeding reaction between the SOD1 aggregated form and normal SOD1, altering nucleation probability and the generation of toxic species.

### KINETICS OF NEURONAL CELL LOSS

A simple analytical theory has been proposed to account for the lag time distribution for a stochastic nucleated polymerization reaction in which the lag time of nucleation probability versus the number of unaffected cells fits an exponential distribution (Szabo, 1988). Consistent with this exponential decay in the number of unaffected cells, Colby et al. (2006) showed that, in cultured striatal neurons expressing the mutant Huntington’s disease (HD) protein, the probability of a cell remaining aggregate-free dropped exponentially with time.

If soluble oligomers implicated in the toxicity of several neurodegenerative disorders are formed via on-pathway nucleation-dependent polymerization, the probability of nucleation would be considered to pose a constant risk for cell death regardless of its toxicity (Perutz and Windle, 2001; Sugaya and Matsubara, 2009). Nucleation is a rare event that occurs randomly in time and space, and is a rate-limiting process with a thermodynamically unfavorable state (Perutz and Windle, 2001). In a constant-risk model of neurodegeneration, the death of a neuron is initiated randomly in time by a series of single, rare catastrophic events, and the death of any given cell is independent of that of any other cell (Clarke et al., 2000). Under the constant-risk assumption, the kinetics of neuronal loss over time exhibits an exponential function (**Figure 2C**, left panel; Clarke et al., 2000). Accordingly, the kinetics of cell death would be expected to show an exponential function, with risk decreasing as further elongation of aggregates reduced the generation of toxic, soluble oligomers; thus, risk would decrease in proportion to the extent to which aggregation increased (**Figure 2C**, middle panel). Under the scenario of the failure to reduce the concentration of unfolded mutant SOD1 monomers by cross-seeding aggregates of normal SOD1 monomers, we would expect the kinetics of cell death to closely fit an exponential function, but in the opposite direction (**Figure 2C**, right panel).

### ASSOCIATION WITH CLINICAL FINDINGS

A motor pool refers to all of the individual motor neurons that innervate a single muscle. Because of motor pools in the spinal cord are clustered in distinct columns of motor neurons extending over multiple spinal cord segments, a longitudinal study for estimating the number of motor units from individual muscles in ALS patients may provide a mechanistic insight into local disease spread. A number of techniques have been developed to estimate the number of motor units in humans by defining a motor unit as the spinal motor neuron and its axon together with the muscle fibers it innervates. Motor unit number estimation (MUNE) is a technique that uses EMG to estimate the number of motor units in a muscle. Using the most reliable electrophysiological method for MUNE, the exponential nature of lower motor neuron loss over time in both ALS patients and SOD1-linked FALS patients has recently been demonstrated in all of the muscles examined (Baumann et al., 2010, 2012). Furthermore, MUNE values obtained with the Bayesian method show a solid correlation with the actual number of lower motor neurons in the spinal cord, determined by histology (Ngo et al., 2012). The exponential kinetics of neuronal cell loss is consistent with the “one-hit” model of neurodegeneration described by Clarke et al. (2000). Thus, the endogenous, stochastic occurrence of one-hit events in a homogenous population of lower motor neurons may play a pivotal role in local disease spread. The alternative view of prion-like propagation in the context of the protective-aggregation hypothesis may well explain the exponential nature of lower motor neuron loss in SOD1-linked FALS.

### ASSOCIATION WITH TRANSGENIC MOUSE MODEL

Further evidence to support the prognostic role of prion-like propagation has come from co-expression studies using transgenic mice. Since most familial ALS patients with SOD1 mutations are heterozygous, recent studies have utilized transgenic mice expressing both human WT (hWT) and ALS-related mutant SOD1 to more accurately recapitulate SOD1 behavior *in vivo*. Co-expression of SOD1^hWT^ exacerbates the disease phenotypes of SOD1^G93A^ (Jaarsma et al., 2000; Fukada et al., 2001), SOD1^G85R^ (Wang et al., 2009), and SOD1^A4V^ mice (Deng et al., 2006), hastening the appearance of cellular pathologies and shortening survival times. The effect of the WT protein on SOD1^A4V^ mice is particularly dramatic; even though ALS patients with this mutation exhibit particularly rapid disease progression, mice expressing SOD1^A4V^ alone do not develop motor neuron disease within their lifetimes (Gurney et al., 1994; Jaarsma et al., 2000). The alternative view of prion-like propagation suggests that normal SOD1 prevents the reduction in the concentration of mutant SOD1 monomers, with a predicted shorter survival (**Figure 2**). Indeed, the concentration of monomeric G93A-SOD1 protein is markedly elevated in the tissues of transgenic mice carrying both G93A-SOD1 and hWT-SOD1 genes (G93A/hWT-mice) compared to that in G93A-mice (Fukada et al., 2001). Furthermore, in the affected tissue, SOD1 aggregates containing both WT and mutant protein have been observed (Deng et al., 2006; Wang et al., 2009), suggesting that hWT-SOD1 is “recruited” into non-native oligomers by the mutant SOD1, possibly by the cross-seeding reaction.

## GENOTYPE–PHENOTYPE CORRELATIONS IN SOD1-LINKED FALS UNDER THE PROTECTIVE-AGGREGATION HYPOTHESIS

The nucleation aggregation theory predicts that the probability of nucleation is an exponential function of the free energy of nuclear formation (Perutz and Windle, 2001). In the case of HD caused by the expansion of CAG trinucleotide repeats, which creates proteins containing long, toxic polyglutamine repeats, the change in free energy per additional glutamine repeat is expected to be constant (Perutz et al., 2002). Therefore, the probability of nucleation is expected to rise exponentially with the number of repeats. This appears to be reflected by the exponential relationship between the extent of expansion and age of onset in HD (Perutz and Windle, 2001; Sugaya et al., 2007; Sugaya and Matsubara, 2009). In the case of SOD1, a thermodynamic analysis suggests that the WT protein is a so-called three-state dimer in which the individual monomers also adopt their correct folded structures in the absence of an intermolecular interface or stabilizing Zn and Cu ions (Banci et al., 1998; Stroppolo et al., 2000; Strange et al., 2003). Several recent studies have implicated such immature monomeric SOD1 species as precursors in the ALS mechanism (Furukawa et al., 2004; Hough et al., 2004; Schmidlin et al., 2009). Reduced stability shifts the folding equilibrium towards denatured monomers.

There are differences in the ability of amyloid conformers with different SOD1 mutations to cross-seed with normal SOD1 (Chia et al., 2010; Hwang et al., 2010). Using the altered free energy of the denatured monomer of SOD1 species with different mutations as an index, we examined whether the prognostic role of prion-like propagation can explain the variance in survival times among patients with different SOD1 mutations.

### CHANGES IN THE STABILITY OF SOD1 MUTANT PROTEINS

Native SOD1 is an extremely stable, obligate homodimer. A key event in SOD1-linked ALS seems to be the pathological formation of toxic species of misfolded SOD1 as a result of initially unfolded SOD1 mutants. Dimeric apoSOD1^pWT^ (metal depleted, pseudo-WT SOD1 with Cys-to-Ala substitutions at positions 6 and 111) has been shown to exhibit three-state folding behavior in which the monomer folds independently following a classical two-state process (Lindberg et al., 2004). After folding, the structured monomers assemble as homo-dimers according to the relationship,

$$2D⇄2M⇄M_{2},$$

where *D* is the unfolded monomer, *M* is the folded monomer, and *M*_2_ is the dimer. Equilibrium studies tell us about the difference in free energy between the folded and the denatured state. Using the stopped-flow technique, Lindberg et al. (2004, 2005) reported the effect of protein destabilization (ΔG) upon ALS-associated point mutations in SOD1 (Byström et al., 2010). Using these date, we performed a regression analysis on the relationship between mean age at disease onset (or respiratory failure death) in patients with different point mutations and the effects of each point mutation on protein destabilization, applying a mathematical model that assumes that toxic species of the misfolded SOD1 are formed via on-pathway nucleation-dependent polymerization:

Dimer⇄Unfolded⁢monomer⇄Nucleus→Fibril

The clinical data used in this study and protein stability changes (ΔG) for each SOD1 mutation are summarized in Supplemental Table 1. Protein stability changes (ΔG) were normalized for comparison purposes.

### MATHEMATICAL MODEL OF THE PROTECTIVE-AGGREGATION HYPOTHESIS BASED ON NUCLEATION-DEPENDENT POLYMERIZATION

An exponential relationship would be expected to hold between the effect of protein destabilization (ΔG) and the nucleation lag time of SOD1 aggregation, similar to the relationship between polyQ length and nucleation lag time of polyQ aggregation (Sugaya and Matsubara, 2009, 2012). If the nucleation rate over time acts as a constant risk for neuronal cell damage in a homogenous cell population, the probability of aggregate-free neurons (or the proportion of intact neurons) as a function of time* t* would be expected to decline exponentially, consistent with the stochastic appearance of nucleation. The rate constants for nucleation, estimated by non-linear least-squares algorithms, are ∼10,000,000 times smaller than those for fibril growth (Lee et al., 2007). Therefore, under the assumption that toxic species of misfolded SOD1 are formed via on-pathway nucleation-dependent polymerization, the proportion of intact neurons at time* t* is largely determined by two integral elements: one reflects the probability distribution function for nucleation lag time (*t*_N_), and the other corresponds to an extension time (*t*_E_), reflecting the protective effects of aggregate growth attributable to the decreased risk of neuronal cell loss. Their relationships can be described by* t*^2^ = *t*_N_^2^ + *t*_E_^2^ in a linear regression model. We initially made the assumption that the extent of motor neuron loss at the site of onset would be nearly identical at disease onset regardless of the nature of the SOD1 mutation. Then, mean age at onset (*t*_A_) can be expressed as a function of protein stability change (*x*):

(1) $${\left(mean\,{t_{A}}^{2}-{t_{EA}}^{2}\right)}^{1/2}=t_{NA}=f\left(x\right)$$

where *t*_EA_ is *t*_E_ at disease onset, *t*_NA_ is* t*_N_ at disease onset, and *f*(*x*) is the integration of the probability distribution function of nucleation lag time at disease onset as an exponential function of the change in protein stability. Similarly, mean age at respiratory failure death (*t*_R_) can be expressed as

(2) $${\left(mean\,{t_{R}}^{2}-{t_{ER}}^{2}\right)}^{1/2}=t_{NR}=g\left(x\right)$$

where* t*_ER_ is *t*_E_ at respiratory failure death, *t*_NR_ is* t*_N_ at respiratory failure death, and* g*(*x*) is the integration of the probability distribution function of nucleation lag time at respiratory failure death as an exponential function of the change in protein stability (*x*). Therefore, mean survival time among subjects with different SOD1 mutations can be expressed as a function of protein stability change as

(3) $$mean\,t_{R}-mean\,t_{A}={\left({t_{ER}}^{2}+{\left[g\left(x\right)\right]}^{2}\right)}^{1/2}-{\left({t_{EA}}^{2}+{\left[f\left(x\right)\right]}^{2}\right)}^{1/2}$$

We employed linear regression with logarithmic transformation of Eqs 1 and 2, thus invoking an intrinsically linear model. Linear regression analysis was then applied to determine* t*_E_ values by identifying the points at which *R*^2^ values were identical for a quadratic curve and a linear model that best fit a linear relationship. Models were evaluated using *R*^2^ values, the *F*-test, and analyses of residual error to test whether the assumptions of the regression were reasonably satisfied.

### PRION-LIKE PROPAGATION IS REFLECTED IN VARIABILITY OF SURVIVAL TIME

**FIGURE 3:**
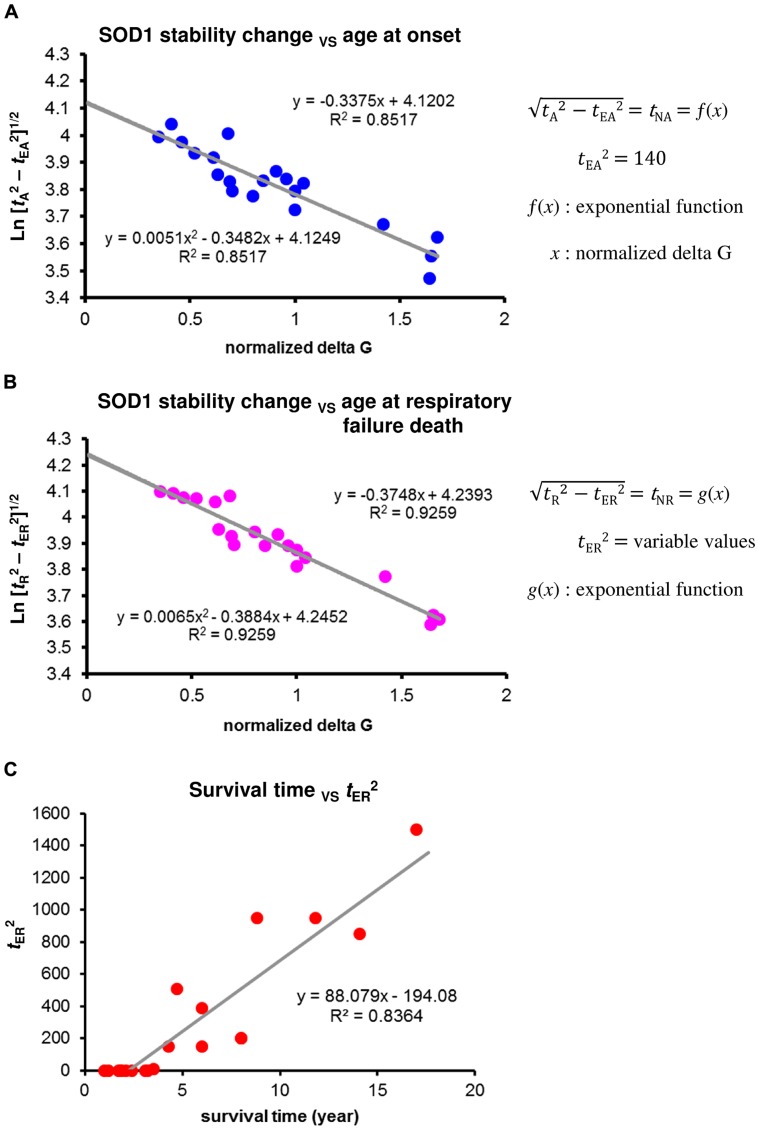
**Changes in the stability of SOD1 proteins and phenotypic variability of SOD1-linked FALS. (A)** Correlation between mean age at onset among patients with different SOD1 mutations and changes in the stability of mutant SOD1 protein. A linear regression analysis using a logarithmic transformation of Eq. 1 provided the best fit to a linear model when *t*_EA_*^2^* = 140. The *t*_EA_^2^ value was determined by identifying the points at which *R*^2^ values were identical for a quadratic curve and a linear model. **(B)** Correlation between mean age at respiratory failure death among patients with different SOD1 mutations and changes in the stability of mutant SOD1 protein. A linear regression analysis using a logarithmic transformation of Eq. 2 provided the best fit to a linear model when the values of *t*_ER_^2^ were as shown in Supplementary Table 1 (variable values of *t*_ER_^2^). Each value of *t*_ER_^2^ was determined by identifying the point at which the *R*^2^ value was identical for a quadratic curve and a linear model. **(C)** Correlation between the values of *t*_ER_^2^ from the result of **(B)** and mean survival time among patients with different SOD1 mutations.

The relationship between mean age at onset in patients with different SOD1 mutations and changes in the stability of their SOD1 mutant proteins showed a good fit with a linear regression model of logarithmically transformed Eq. 1 (*R*^2^ = 0.85, *F* = 126.0) when *t*_EA_^2^ = 140 (**Figure 3A**). The variability of age at onset is well fit by the model when the values of *t*_EA_^2^ are identical as expected by an exponential decline of neuronal cell loss. Accordingly, the alternative role of prion-like propagation, together with Eq. 3, suggests that the variability in mean survival time among patients with different SOD1 mutations is largely dependent on the differences in extension time at respiratory failure death (*t*_ER_). The concentration of unfolded mutant SOD1 monomers varies according to the efficacy of the cross-seed reaction between the SOD1 aggregated form and normal SOD1. Because the nucleation process is concentration dependent, this alters the probability of nucleation and subsequent generation of toxic species of misfolded SOD1, leading to the variable values of *t*_E_^2^ (extension time due to the protective effects of aggregate growth versus toxic species of misfolded SOD1) from short survival to long survival. We first considered that the values of *t*_ER_ are nearly identical in subjects with SOD1 mutations that show a mean survival time less than 2.5 years, and determined *t*_ER_ value using the regression model of logarithmically transformed Eq. 2 to provide the best fit to a linear model. In a similar way, we next determined each value of *t*_ER_ in patients with different SOD1 mutations. The relationship between mean age at respiratory failure death in patients with different SOD1 mutations and changes in the stability of their mutant SOD1 proteins showed a good fit with a linear regression model of logarithmically transformed Eq. 2 (*R*^2^ = 0.93, *F* = 172.1) for the variable *t*_ER_^2^ values shown in Supplementary Table 1 (**Figure 3B**). Remarkably, the values of *t*_ER_^2^ accounted for 84% of the variability of mean survival time in patients with different SOD1 mutations (**Figure 3C**). However, the values of *t*_ER_^2^ showed no significant correlation with the aggregation propensity of individual SOD1 mutants (Vassall et al., 2011). Although data are available for only a limited number of SOD1 mutants (Chia et al., 2010; Hwang et al., 2010), the results appear to be consistent with differences in the ability of SOD1 amyloid conformers with different mutations to cross-seed with normal SOD1.

## DISCUSSION

In inherited neurodegenerative disorders, delayed clinical onset, in which symptoms may not appear for years or decades, is often assumed to reflect the occurrence of age-dependent cumulative damage. One prediction of the cumulative-damage hypothesis is that the probability of cell death will increase over time, and the kinetics of neuronal death over time can be expressed by a sigmoidal function. However, Clarke et al. (2000) reported that the kinetics of neuronal loss in many forms of neurodegeneration appeared to be exponential, and in fact could be better explained by a mathematical model in which the risk of cell death remains constant (one-hit model). The exponential decimation of remaining lower motor neurons over time in ALS patients is also consistent with the one-hit model of neurodegeneration (Baumann et al., 2010, 2012). Furthermore, Clarke and Lumsden (2005) showed that the one-hit model of neurodegeneration can be improved using stretched exponential decay models, which most easily fit data in which the rate of death decreases over time, consistent with multiple populations of neurons coexisting, each with a different constant risk of death. However, there are some scenarios in which it may also be possible to explain such kinetics as an exponentially decreasing risk of neurodegeneration. For example, if dying neurons released a cyto-protective substance such as a neurotrophic factor into their environment, then the concentration of that factor would increase as more neurons were affected, causing a concomitant decline in the risk of cell death. One scenario has also emerged in the context of the protective-aggregation hypothesis in which toxic species of misfolded proteins, as soluble oligomers, are formed as an on-pathway intermediate in nucleation-dependent polymerization. The expectation under this scenario is that the kinetics of cell death would reveal an exponentially decreasing risk, with further fibril elongation reducing the risk posed by soluble oligomers in proportion to the increased extent of aggregates. These scenarios suggest that, although initiation of the neurodegenerative process occurs randomly in time as a series of independent events for each neuron, under the condition of exponentially decreasing risk, the progression of the neurodegenerative process may be influenced by other cells.

One major unresolved question regarding the molecular mechanism underlying neurodegenerative process in patients with SOD1-linked FALS is why the prognoses are so varied despite a pathogenic mechanism in common. The duration of illness is relatively consistent for each SOD1 mutation, but is variable among different mutations. Wang et al. (2008) reported that two synergistic properties, increased protein aggregation propensity and decreased protein stability, account for 69% of the variability in mutant Cu/Zn-superoxide-dismutase-linked familial ALS patient survival times (Wang et al., 2008). However, in their regression analysis, about half of the patients had A4V mutation. Its corrected value account for about 20% of the variability in survival times among subjects with different mutation. Recently, Vassall et al. (2011) reported that in their depth analyses, there is minimal to no correlation between observed aggregation, predicted aggregation propensity, and disease duration. Thus, the major factor, which contributes to the disease progression, is still unknown. The correlation between polyglutamine repeat length and the effects of repeat length on the rate of disease progression, as well as age of onset, were well explained by the regression models of Eqs 1 and 2 when polyglutamine repeat length was used as an index (*x*; Sugaya and Matsubara, 2009, 2012). However, one distinct feature in the results of SOD1-linked FALS stands out: the extension time (*t*_E_) is nearly identical among subjects with different polyglutamine repeat-length, even those with late-stage HD (Sugaya and Matsubara, 2012). Compared with the mechanisms of prion-like propagation in SOD1 aggregates, one clear difference is that mutant huntingtin aggregates do not cross-seed* in vitro* with huntingtin containing normal lengths of polyglutamine tracts (Busch et al., 2003). These features support the idea that differences in the ability of SOD1 amyloid conformers with different mutations to cross-seed with normal SOD1 is reflected in the variability of survival time. These findings, together with the results of **Figure 3** suggest the prognostic value of prion-like propagation under the protective-aggregation hypothesis, showing that it may account for up to 84% of the variability in survival times across subjects with different SOD1 mutations.

## CONCLUSION

At first glance, several major aspects of the pathogenesis of SOD1-linked ALS, including cell-autonomous and non-cell-autonomous processes, prion-like propagation and exacerbation of disease phenotypes in transgenic mice expressing both WT and mutant SOD1, appear to be independent events, like pieces of a puzzle. Viewed in the context of the protective-aggregation hypothesis, the molecular events in prion propagation raise the possibility of “prion-like propagation,” providing an alternative disease model to account for the mechanism of SOD1-linked ALS. Considerable work remains to verify this disease model; however, a central issue for future studies is connecting the factors involved in the pathogenesis of SOD1-linked FALS with each other to delineate the prognosis and spread of the disease.

## Conflict of Interest Statement

The authors declare that the research was conducted in the absence of any commercial or financial relationships that could be construed as a potential conflict of interest.
