# Supplementary material for: Prognostic role of “prion-like propagation” in SOD1-linked familial ALS: an alternative view
Source: Front Cell Neurosci. 2014 Oct 31;8:359. doi: 10.3389/fncel.2014.00359 (PMC4215625; doi:10.3389/fncel.2014.00359)
Supplement: Supplementary file 1 [file Data_Sheet_1.DOCX]

**Supplemental Table 1**

**Clinical features and changes in the stability of ALS-associated SOD1 mutants**

| Genotype | Mean age at onset  (years) | Mean age at RFD  (years) | Survival time  (years) | Number of patients | Normalized ΔG | Estimated unit of nucleus* | Estimated *t*_ED_^2^ value |
| --- | --- | --- | --- | --- | --- | --- | --- |
| A4V | 47.7 | 48.9 | 1.2 | 205 | 0.85 | 1 | 0 |
| V7E** | 48.5 | 54.2 | 5.7 | 3 | 0.52 | 1 | ― |
| G37R | 36.9 | 53.9 | 17 | 27 | 0.55 | 3 | 1500 |
| L38V | 41.1 | 43.5 | 2.4 | 22 | 0.71 | 2 | 0 |
| G41D | 45.2 | 59.3 | 14.1 | 15 | 0.8 | 1 | 850 |
| G41S | 47.9 | 48.9 | 1 | 16 | 0.96 | 1 | 0 |
| H43R | 49.3 | 51.1 | 1.8 | 12 | 0.91 | 1 | 0 |
| H46R** | 45.6 | 63.2 | 17.6 | 49 | 0.03 | ― | ― |
| H48Q** | 43.6 | 44.8 | 1.2 | 4 | 0.45 | 2 | ― |
| D76V** | 46 | 64.8 | 18.8 | 4 | 0.15 | 3 | ― |
| D76Y** | 55 | 64 | 9 | 2 | 0.16 | 2 | ― |
| L84V | 47.6 | 50.8 | 3.2 | 10 | 0.69 | 1 | 0 |
| G85R | 55.5 | 61.5 | 6 | 11 | 0.35 | 1 | 150 |
| N86D** | NA | NA | NA | 0 | 0.32 | ― | ― |
| N86K | 58.2 | 59.9 | 1.7 | 7 | 0.41 | 1 | 0 |
| N86S** | 47.8 | 54.6 | 6.8 | 4 | 0.28 | 2 | ― |
| D90A | 52.4 | 60.4 | 8 | 15 | 0.26 | 2 | 200 |
| D90V** | 46 | 48.7 | 2.7 | 3 | 0.49 | 2 | ― |
| G93A | 46 | 49.1 | 3.1 | 16 | 0.7 | 1 | 0 |
| G93D | 39.3 | 48.1 | 8.8 | 7 | 0.84 | 2 | 950 |
| G93R** | 35 | 40.3 | 5.3 | 4 | 0.6 | 2 | ― |
| G93S | 51.6 | 59.6 | 8 | 11 | 0.61 | 1 | 200 |
| G93V | 46 | 52 | 6 | 12 | 1 | 1 | 390 |
| E100G | 47.3 | 52 | 4.7 | 50 | 0.52 | 2 | 510 |
| E100K** | NA | NA | NA | 1 | 0.3 | ― | ― |
| D101G** | 38 | 39.9 | 1.9 | 3 | 0.4 | 2 | ― |
| D101N** | NA | 42.4 | NA | 17 | 0.1 | ― | ― |
| I104F** | 33 | 54.3 | 21.3 | 3 | 0.37 | ― | ― |
| S105L | 48.7 | 52.2 | 3.5 | 7 | 0.63 | 1 | 10 |
| L106V | 34.3 | 36.2 | 1.9 | 6 | 0.82 | 2 | 0 |
| I113T | 56.3 | 60.6 | 4.3 | 38 | 0.68 | 1 | 150 |
| G114A** | 37.5 | 40.2 | 2.7 | 2 | 0.76 | 2 | ― |
| D124V** | 42 | NA | NA | 0 | 0.01 | ― | ― |
| D125H** | 65 | 66.8 | 1.8 | 2 | 0.21 | 1 | ― |
| S134N** | 51.3 | 52.5 | 1.2 | 3 | 0.23 | 1 | ― |
| N139D** | NA | NA | NA | NA | 0.07 | ― | ― |
| N139K** | NA | NA | NA | NA | 0.24 | ― | ― |
| L144F | 54.6 | 66.4 | 11.8 | 15 | 0.46 | 1 | 950 |
| L144S** | 42.5 | 54.8 | 12.3 | 2 | 0.36 | 3 | ― |
| V148G | 43.1 | 45.2 | 2.1 | 11 | 1 | 1 | 0 |

RFD, respiratory failure death; NA, not available. *Number of monomeric units comprising the nucleus was estimated using the regression model of Eq. 1, with the unit of the nucleus in A4V aggregates defined as one. **SOD1 mutations found in fewer than five patients were excluded from the regression analysis. H46R was also excluded from the analysis because it showed little difference in protein stability compared with wild-type SOD1, suggesting that additional mechanisms play a significant role in disease pathogenesis. Data were derived from previously reported studies [1-3].

References

1. Lindberg MJ, Byström R, Boknäs N, et al. (2005). Systematically perturbed folding patterns of amyotrophic lateral sclerosis (ALS)-associated SOD1 mutants. *Proc Natl Acad Sci USA*, 102, 9754-9759.

2. Byström R, Andersen PM, Gröbner G, Oliveberg M. (2010). SOD1 mutations targeting surface hydrogen bonds promote amyotrophic lateral sclerosis without reducing apo-state stability. *J Biol Chem*, 285, 19544-19552.

3. Wang Q, Johnson JL, Agar NY, Agar JN. (2008). Protein aggregation and protein instability govern familial amyotrophic lateral sclerosis patient survival. *PLoS Biol*, 6, e170.
